# The Effects of Repeat-Dose Doxorubicin on Cardiovascular Functional Endpoints and Biomarkers in the Telemetry-Equipped Cynomolgus Monkey

**DOI:** 10.3389/fcvm.2021.587149

**Published:** 2021-02-23

**Authors:** Michael J. Engwall, Nancy Everds, James R. Turk, Hugo M. Vargas

**Affiliations:** ^1^Translational Safety and Bioanalytical Sciences, Amgen Inc., Thousand Oaks, CA, United States; ^2^Non-clinical Sciences, Seattle Genetics, Inc., Seattle, WA, United States

**Keywords:** doxorubicin, macaca fascicularis, heart failure, QTc interval prolongation, echocardiography, telemetry, troponin

## Abstract

**Purpose:** Doxorubicin-related heart failure has been recognized as a serious complication of cancer chemotherapy. This paper describes a cardiovascular safety pharmacology study with chronic dosing of doxorubicin in a non-human primate model designed to characterize the onset and magnitude of left ventricular dysfunction (LVD) using invasive and non-invasive methods.

**Methods:** Cynomolgus monkeys (*N* = 12) were given repeated intravenous injections of doxorubicin over 135 days (19 weeks) with dosing holidays when there was evidence of significantly decreased hematopoiesis; a separate group (*N* = 12) received vehicle. Arterial and left ventricular pressure telemetry and cardiac imaging by echocardiography allowed regular hemodynamic assessments and determination of LVD. Blood samples were collected for hematology, clinical chemistry, and assessment of cardiac troponin (cTnI) and N-terminal pro b-type natriuretic peptide (NT-proBNP). Myocardial histopathology was a terminal endpoint.

**Results:** There was variable sensitivity to the onset of treatment effects, for example 25% of doxorubicin-treated animals exhibited LVD (e.g., decreases in ejection fraction) following 50–63 days (cumulative dose: 8–9 mg/kg) on study. All animals deteriorated into heart failure with additional dosing 135 days (total cumulative dose: 11–17 mg/kg). Reductions in arterial pressure and cardiac contractility, as well as QTc interval prolongation, was evident following doxorubicin-treatment. Both cTnI and NT-proBNP were inconsistently higher at the end of the study in animals with LVD. Measurements collected from control animals were consistent and stable over the same time frame. Minimal to mild, multifocal, vacuolar degeneration of cardiomyocytes was observed in 7 of 12 animals receiving doxorubicin and 0 of 12 animals receiving vehicle.

**Conclusions:** This repeat-dose study of doxorubicin treatment in the cynomolgus monkey demonstrated a clinically relevant pattern of progressive heart failure. Importantly, the study revealed how both telemetry and non-invasive echocardiography measurements could track the gradual onset of LVD.

## Introduction

Doxorubicin (Adriamycin) has been a common treatment for cancer since its introduction in the 1960s. It is well-known that treatment is associated with cardiotoxicity which can progress to congestive heart failure (CHF) (1). This concern limits the use of doxorubicin in human patients. An early paper stated the core concerning issue: “*The morbidity and mortality rates related to anthracycline-induced cardiotoxicity will continue to increase because of several factors: significant cardiac injury, which occurs even with low-dose therapy and commonly occurs with therapeutic doses; the use of monitoring techniques that are often insensitive to subclinical cardiac damage; lack of effective cardio protection; and late-onset ventricular impairment occurring in asymptomatic patients years after uncomplicated anthracycline treatment*” (2). These concerns persist to this day.

The mechanism underlying the cardiovascular toxicity may be related to the generation of reactive oxygen species (ROS) but it is well-appreciated that the toxicity is multifactorial and includes effects stemming from mitochondrial impairment and disruptions of myocyte calcium handling (3–5). The toxicity is also strongly linked to the generation of secondary metabolites, which can vary among the anthracyclines (6). More recently, there has been a growing concern with delayed-onset degradation of cardiac function (8). While the damage to the heart is thought to be immediate, a patient's compensatory mechanisms may delay the onset of clinically detectable changes for years (7). With lifetime doxorubicin doses of 400–450 mg/m^2^, a heart failure incidence of 5% (10% in older patients) can be expected (8). Additionally, the combination of doxorubicin with other cancer therapies (e.g., trastuzumab) augments the risk of cardiotoxicity (9).

While clinical diagnostic tests are poor predictors of long-term prognosis, they are used to diagnose and document the extent of functional changes as cardiac injury progresses. ECG changes such as QTc prolongation emerge during development of sequelae to treatment (10). Likewise, measurements of cardiac function derived from echocardiography such as ejection fraction and fractional shortening as well as indices of diastolic function are decreased in the patients who develop heart disease following treatment with doxorubicin (11). Recent papers from a consortium of pharmaceutical companies highlighted the utility of measurements of left ventricular endpoints (e.g., dP/dt_max_, an index of cardiac contractility) as a biomarker of cardiac function (12, 13).

The clinical manifestation of doxorubicin-related cardiotoxicity has been modeled in several; preclinical species such as rats, mice, rabbits, and dogs (14–18). The dosages and dosing intervals used varied considerably across the studies. With regard to higher species like the non-human primate, three evaluations of doxorubicin were conducted. One early study used cynomolgus monkeys treated with doxorubicin (1 mg/kg monthly) for 26 months (19). Although clinical signs of congestive heart failure were observed in the majority (8/10) of animals, no quantitative measurements of cardiac function were collected. In a second study (reported in abstract form), a different doxorubicin dose regimen (2 mg/kg/week for 3 weeks followed by 1 mg/kg/2 weeks until evidence of cardiac dysfunction occurred) was used in cynomolgus monkeys, although the magnitude of echocardiography changes was not stated (20). Lastly, Takayama et al. (21) demonstrated that doxorubicin (1 mg/kg/every 4 weeks for 114 months) administration caused myocardial structural changes related to heart failure, but no functional measurements were collected from those animals.

Given the important role and value of non-human primate in cardiovascular safety assessments for new therapeutics (22), and the lack of nonclinical information on the sensitivity of non-human primate to doxorubicin-induced cardiovascular toxicity, we characterized the effect of this clinically relevant anticancer therapeutic in the cynomolgus monkey to better understand the translational value of this animal model. In this study, serial measures of cardiac function were examined in cynomolgus monkeys given repeated doses of Dox. Assessments included telemetry collection of electrocardiograms, hemodynamics, circulating levels of cTnI and echocardiography. The study used a dose regimen based on prior literature that was intended to produce progressive decreases in left ventricular systolic function while endeavoring to avoid non-cardiovascular toxicities (e.g., leukopenia) also associated with doxorubicin treatment.

## Methods

The effects of doxorubicin treatment on measures of cardiac function were characterized in cynomolgus monkeys over an observation period of up to 135 days. The study was performed at Covance Laboratories, Inc. (Madison, WI).

### Animal Care and Treatment

Male cynomolgus monkeys (*Macaca fascicularis*) of Chinese origin obtained from Covance Research Products, Inc. (2–12 years old; 3–12 kg), were housed in stainless steel cages. Animals selected for this study were based on age (planned retirement from the colony) and expiring telemetry battery life. Care and use of animals was as specified by United States Department of Agriculture Animal Welfare Act (9 CFR, Parts 1, 2, and 3) and as described in the Guide for the Care and Use of Laboratory Animals (23). Animals were individually or socially housed indoors in species-specific housing at an Association for Assessment and Accreditation of Laboratory Animal Care-accredited facility. All research protocols were approved by the Covance Institutional Animal Care and Use Committee (IACUC) prior to dose administration. Certified Primate Diet (#5048, PMI, Richmond, IN) was provided daily in amounts appropriate for the age and size of the animals. Animals had *ad libitum* access to water (automatic watering system or bottle), were maintained in standard housing conditions (12:12 h light:dark cycle; 20° −26°C; 30–70% humidity) and had enrichment opportunities, e.g., food treats, fresh fruit, toys, and social interaction. A veterinary medical treatment plan was developed (pre-study) to provide supportive care based on clinical observations, clinical pathology data, hemodynamic findings, and/or echocardiography findings, including the management of pain and suffering (euthanasia). Palliative and prophylactic procedures were based upon consensus agreement between the study director and attending laboratory animal veterinarian. Terminal procedures included scheduled (day 135; last day of study) or unscheduled (due to morbidity or poor health) euthanasia by deep anesthesia by ketamine/sodium pentobarbital injection and exsanguination. Final blood samples (vena cava) were collected on all animals (when possible) and a complete macroscopic examination was performed at necropsy. Heart tissues (right and left ventricular free walls and ventricular septum) were collected, processed and embedded in paraffin for evaluation by light microscopy.

Animals had been previously instrumented ~1.5 years before the start of the study with telemetry units from Data Science International (DSI). Either D70-PCT (13 animals) or D70-PCTP (11 animals) units were used for collection of electrocardiograms (ECG), body temperature, blood pressure, and left ventricular pressure (LVP) measurements. All animals had been used previously in multiple cardiovascular pharmacology studies but had not been administered any test article for at least 2 weeks prior to study start. The animals used in this study were scheduled for termination due to the duration of their instrumentation.

#### Doxorubicin Dose Regimen

Male animals were randomly divided into vehicle (*N* = 12, average: 6.37 kg) and doxorubicin-treatment (*N* = 12, average: 5.74 kg) groups. The vehicle group contained seven PCT-implanted monkeys (arterial pressure and ECG) and five PCTP-implanted monkeys (arterial and left ventricular pressures and ECG). The doxorubicin group contained six PCT-implanted monkeys and six PCTP-implanted monkeys. Animals were administered weekly intravenous administrations (1 mL/kg) of either sterile saline or 2 (or 1) mg/kg doxorubicin followed by a 2 mL/kg sterile saline flush. Animals administered doxorubicin were given a temporary dosing holiday when neutrophil counts were <1,000/μL. Dosing holidays were also put in place when there was echocardiographic evidence of systolic heart failure (Fractional Shortening ≤ 18%) (Table 1).

#### Telemetry

Data Sciences International (DSI) Dataquest® OpenART® telemetry equipment was used to generate and acquire the data input. This system transferred the data to a PONEMAH Physiology Platform analysis system. The arterial pressure and body temperature raw signal were digitized at a sampling rate of 250 Hz; the left ventricular pressure (LVP) and ECG were digitized at a sampling rate of 500 Hz. Pre-treatment baseline measurements of ECG and blood pressure measurements were collected for a 24-h period ~1 week prior to dosing (study day −6). To avoid procedure related disruption of the cardiovascular data, on-study telemetry data was collected continuously with a logging rate of 60 s from ~46 to 67 h post-treatment (~21 h). Cardiovascular data were monitored on a weekly basis for the remainder of the study (denoted as days 1, 8, 16, 22, 29, 36, 43, 50, 57, 63, 70, 78, 92, 99, 106,113, 120, and 127). Telemetry units failed during the study for 3 animals (I04923 (vehicle) I04937 and I04932 (doxorubicin) on days 30, 37, and 72, respectively. During dosing holidays, data was collected at times approximating those used for dosing days. Telemetric data were reduced into 1-h time bins for analysis. The QT interval was corrected for heart rate (RR interval) using an individual animal correction formula [QTc = QT + [IACF ^*^ (750-RR)], where RR is denoted in msec]. The IACF was derived from a plot of the QT vs. RR interval values collected in at baseline for each animal.

#### Echocardiography

All echocardiography measurements were done with ketamine anesthesia. Principal measurements included left ventricular internal dimensions during diastole and systole, interventricular septal thickness, and left ventricular free wall during diastole from the M-mode. The aortic dimension (diastole) and left atrial dimension (diastole) were measured using a two-dimension image. The primary endpoints used for assessing cardiac function were fractional shortening and ejection fraction and were calculated using the left ventricular measurements. Two baseline measurements (days −4 and −1) of echocardiographic parameters were collected prior to the start of dosing. Echocardiographic endpoints were collected on an approximately biweekly basis for the remainder of the study (days 18, 25, 39, 56, 67, 77, 91, 105, 116, and 127).

#### Blood Sample Analysis

Blood was collected for hematology and clinical chemistry parameters, cardiac biomarkers of acute myocyte damage (cTnI) and heart failure (NT-proBNP), and doxorubicin exposure. Generally, blood was collected from all animals (unanesthetized; femoral vein) twice during the pre-study acclimation period and then at weekly intervals during the treatment phase starting on day 2. Plasma samples were prepared and analyzed in accord to protocol for each endpoint. On four study days, additional blood samples were acquired to measure plasma doxorubicin levels after the initial 2 mg/kg dose (day 1; 8 and 24 h) and following 1 mg/kg (days 36, 78, and 113; 8 and 24 h). The following instruments/methods were used for clinical pathology analysis: hematology: Advia 2120 or 120; coagulation: Stago (Parsippany, NJ); clinical chemistry: Roche Analytics Modular Chemistry Analyzer (Indianapolis, IN); NT-proBNP: ALPCO (human kit BI-20852W) ELISA (Salem, NH); cTnI: Immulite, BeckmanCoulter, Brea, CA.

### Statistical Analysis

Telemetry data were divided into analysis blocks based on photoperiod (light and dark cycles). Circadian changes in heart rate and blood pressure are readily observed in non-human primates and reflect changes in autonomic tone. Data collected by telemetry were analyzed for dosing phase data only. The variance-covariance structure used in the analysis was the one providing the smallest Akaike Information Criterion out of the two: Compound Symmetry and Heterogeneous Compound Symmetry. Because the days were not equally spaced throughout the study period, the Autoregressive (1) and Heterogeneous Autoregressive (1) variance-covariance structures were not investigated. For each analysis block separately, a repeated-measures ANOVA (*Baseline* + *Treatment* + *Day* + *Treatment x Day*) was conducted. *Baseline* was the average for each animal from the pre-dose (Study Day −7) data collection period. If the main effect, *Treatment*, was significant and the *Treatment x Day* interaction was not significant, the data were averaged and analyzed across all the days; if the *Treatment x Day* interaction was significant, the data were analyzed at each day separately. A significance level of 0.05 was used for all testing. Echocardiography data was analyzed separately using a simple two-tailed t-test at for each collection and assuming unequal variance.

## Results

### Doxorubicin Treatment, Tolerability, and Overt Clinical Signs

Over the 135 experimental days, NHP received weekly injections of doxorubicin to induce heart failure, and the control group was given vehicle in the same manner. All doxorubicin-treated animals required dosing holidays (of various durations) due to treatment-related reductions in white blood cell counts (e.g., no treatment on days 22, 29, 43, 57, etc.; Table 1A), an indication of drug-induced bone marrow toxicity. Following three consecutive doses of 2 mg/kg, the dose was lowered to 1 mg/kg for the remainder of the study to minimize bone marrow effects and other clinical effects associated with doxorubicin. This dose modification improved tolerability and all 12 doxorubicin-treated animals survived through study day 100. Plasma levels of doxorubicin were proportionally lower following the dose reduction to 1 mg/kg (Table 1B). The cumulative doxorubicin dose per animal ranged from 11 to 17 mg/kg, or 132–204 mg/m^2^ when expressed per body surface area (Table 2). Following day 100, 10 animals underwent unscheduled euthanasia (*N* = 1 on days 103, 105,107, 109, 114; *N* = 2 on day 123; *N* = 3 on day 130) for moribund condition (4/10) and/or compromised heart function (10/10) (Table 1). All doxorubicin-treated monkeys euthanized at an unscheduled or scheduled interval demonstrated systolic heart failure (e.g., reduced fractional shortening, ejection fraction, and increased left ventricular systolic dimensions).

**Table 1A:** Doxorubicin dosing schedule indicating dosing levels on specific dosing days, dosing holidays, and indication of when animals were euthanized due to deteriorating cardiovascular function.

**NHP#**	**Doxorubicin dose (mg/kg IV) and treatment day**																			
	**1**	**8**	**15**	**22**	**29**	**36**	**43**	**50**	**57**	**63**	**70**	**78**	**86**	**92**	**99**	**106**	**113**	**120**	**127**	**135**
104928	2	2	2			1		1		1	1	1	1	1	1	1	1			
104929	2	2	2			1		1		1		1		1		1		1		
104930	2	2	2			1		1		1	1	1	1	1	1	1	1			
104931	2	2	2			1		1		1	1	1	1	1	1	1	1			
104932	2	2	2			1		1		1	1	1								
104933	2	2	2			1		1		1	1	1	1	1	1	1	1	1		
104934	2	2	2			1		1		1	1	1	1		1					
104935	2	2	2			1		1		1	1	1	1	1	1	1	1	1		
104936	2	2	2			1		1		1	1	1	1	1						
104937	2	2	2			1		1		1	1	1	1	1	1	1	1			
104938	2	2	2			1		1		1	1	1	1	1						
104939	2	2	2			1		1		1	1	1	1	1						

*Gray box: dosing holiday; black box: unscheduled sacrifice. The study concluded on day 135*.

*There were no unscheduled sacrifices in the control group*.

**Table 1B:** Plasma levels of doxorubicin (dox) at 8 and 24 h after intravenous dosing.

	**Day 1**		**Day 36**		**Day 78**		**Day 113**	
	**8 h**	**24 h**	**8 h**	**24 h**	**8 h**	**24 h**	**8 h**	**24 h**
Dox (ng/ml)	31.6 ± 5.7	15.8 ± 3.5	16.5 ± 4.2	8.0 ± 1.6	19.0 ± 5.5	9.1 ± 2.7	16.4 ± 3.5	6.3 ± 1.3

*All values are means ± SD (N = 12; except day 113 was N = 6)*.

**Table 2:** Cumulative dose estimation for each animal treated with doxorubicin.

**NHP#**	**Total dose^a^ mg/kg**	**Mean dosing phase weight^b^ (kg)**	**Total dose^c^ (mg)**	**Dose/body surface area^d^ (mg/m^**2**^)**
104928	16	5.4	86	192
104929	13	4.6	53	156
104930	16	4.9	79	192
104931	16	4.5	72	192
104932	11	5.3	58	132
104933	17	4.5	77	204
104934	13	4.9	63	156
104935	17	5.4	92	204
104936	13	4.7	61	156
104937	16	5.9	94	192
104938	13	8.9	116	156
104939	13	8.8	114	156
Average (SD)	14.5 ± 2.0	5.6 ± 1.6	81 ± 20	174 ± 24

a *Total dose is a sum of each dose (mg/kg) administered to an individual animal during the dosing phase*.

b *Mean body weight was calculated for all body weight intervals in the dosing phase for each individual animal*.

c *Estimated total cumulative dose is the total dose (mg/kg) administered multiplied by the mean body weight (kg) for each individual animal*.

d *The dose per body surface area was determined by multiplying the total dose (mg/kg) administered to individual monkeys by a species-specific mass constant, k_m_. For monkeys, the k_m_ was 12 kg/m^2^ (24)*.

*Cumulative dose levels were dependent on number of doses given which was limited by tolerability*.

Multiple clinical observations were noted for doxorubicin-treated monkeys compared with controls throughout the dosing phase. These included: dry and discolored skin (10/12), low food consumption (12/12), red/orange discolored urine (10/12), vomitus (6/12), and non-formed/liquid/discolored feces (3/12). The overall incidence of effects was relatively low given the number of animals and duration of the study. The observations of dry and/or discolored skin correlated to increased incidence of scabs, broken skin, and sores. In the second half of the study (days 61–135), observations of declining animal health and moribund status included hunched posture, squinted eyes, pale skin, hypoactivity, decreased reactivity to stimuli, labored respirations, recumbent posture, coughing, and feeling cold to touch.

**Figure 1:**
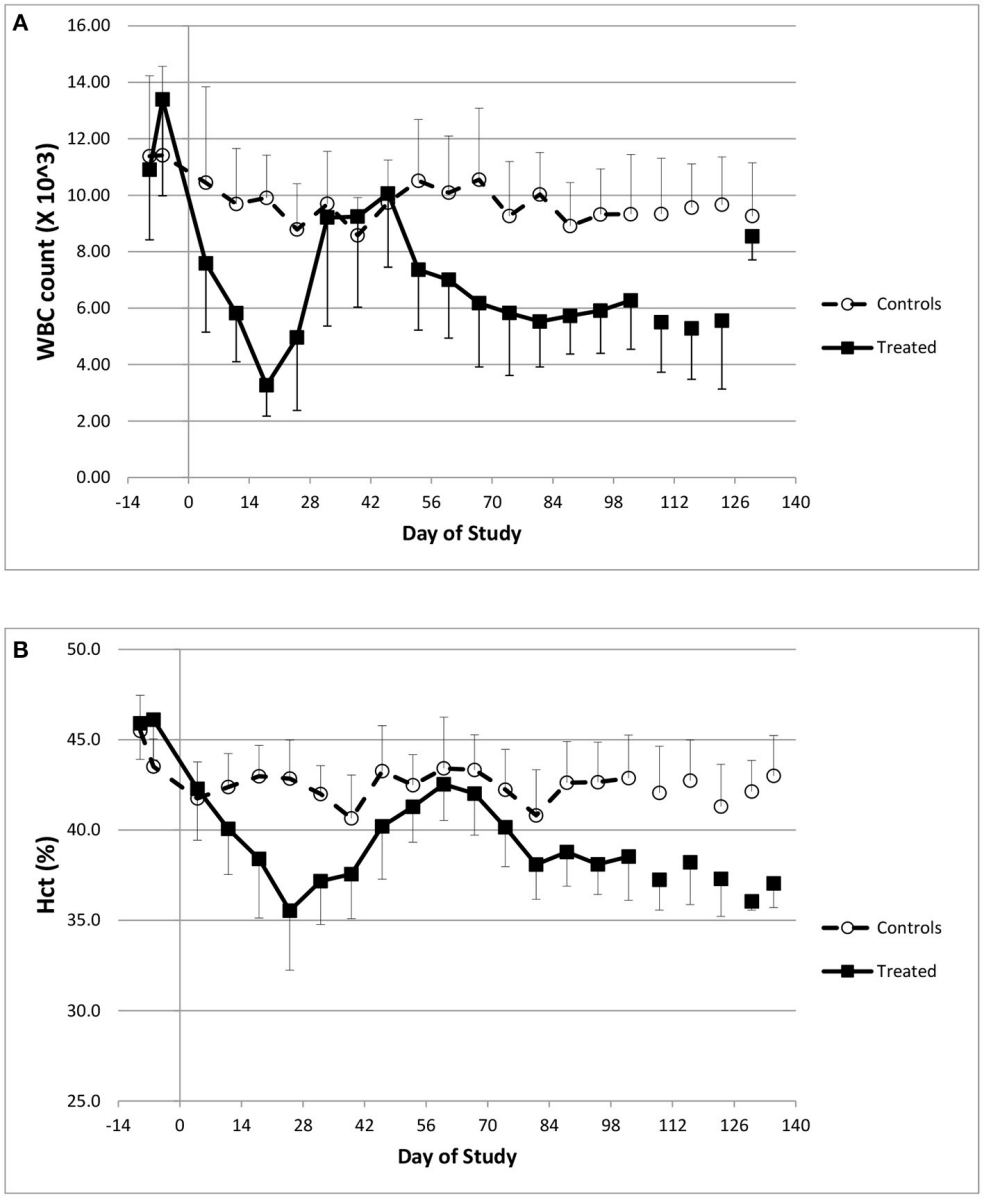
Hematology changes. Changes in white blood cell counts **(A)** and Hematocrit **(B)** over the course of the study.

Hematology data indicated that doxorubicin caused profound reductions in white blood cell counts (Figure 1A), in particular neutrophil levels. At baseline (pre-dose), the normal neutrophil count ranged from 5.1–7.5 × 10^3^/μL, but after three consecutive doses of 2 mg/kg, doxorubicin lowered the average count to 670 × 10^3^/μL (study day 18); the onset of neutropenia triggered the temporary suspension of treatment. In addition, doxorubicin caused minimal to mild reductions in red cell mass (i.e., red blood cell count, hemoglobin concentration), and hematocrit (Figure 1B) during the dosing phase. These hematological effects were attributed to bone marrow dysfunction, were reversible and recovered to near normal levels following the dosing holiday period.

### Onset of Heart Failure: Echocardiography Endpoints

**Figure 2:**
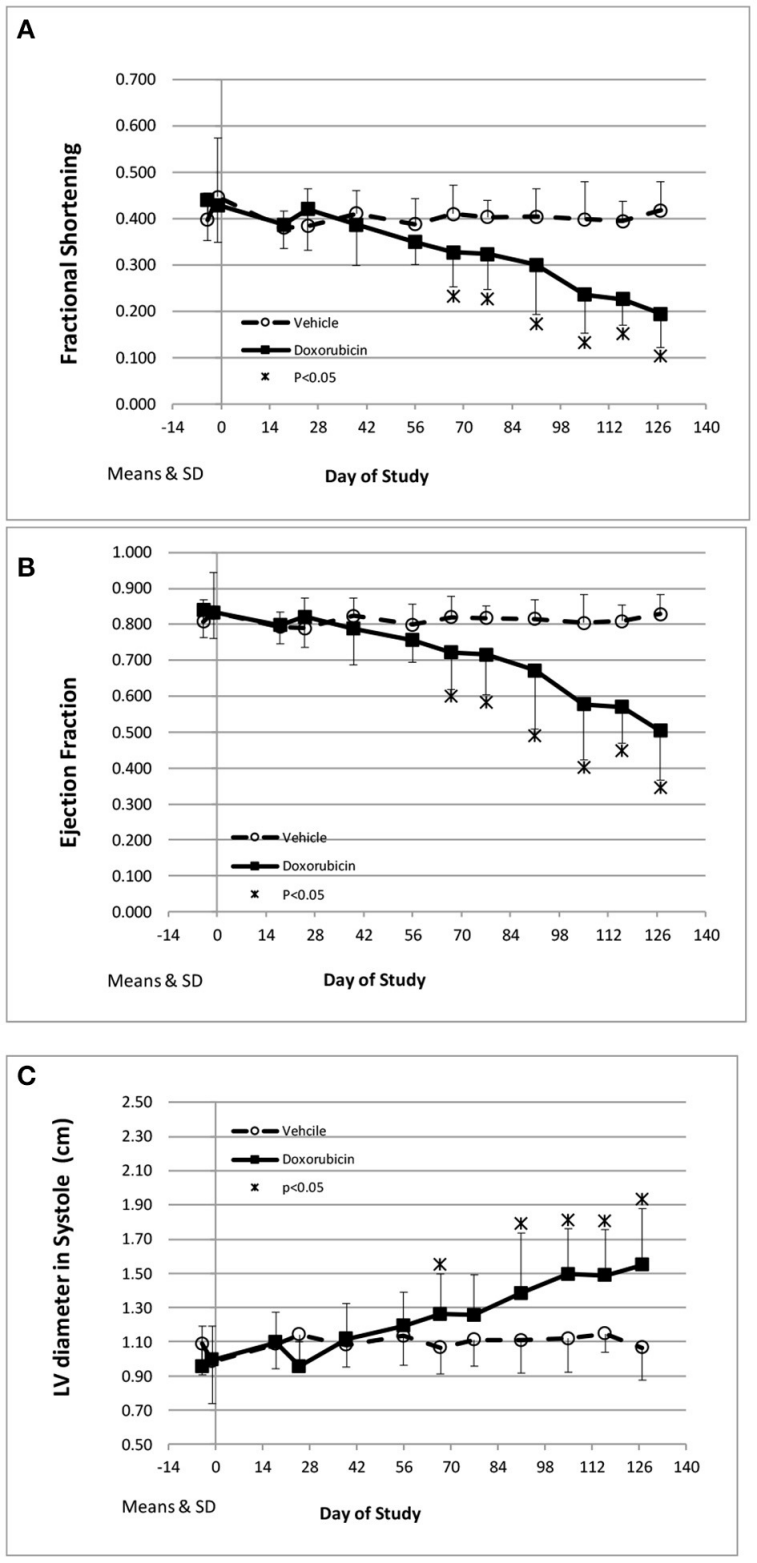
Echocardiography endpoints. Changes in fractional shortening **(A)**, ejection fraction **(B)**, and left ventricular systolic diameter **(C)** over the course of the study. *Statistically different from vehicle treatment.

The key cardiovascular effects of doxorubicin are shown in Table 3 and Figures 2A–C. At baseline, all echocardiographic parameters (FS%, EF%, and left ventricular systolic diameter) were not significantly different. However, the final collected measurements from each animal indicated heart failure as all indices were significantly different in the doxorubicin-treated animals compared to baseline value or compared to vehicle. The earliest echocardiographic alteration associated with doxorubicin was decreased left ventricular systolic function (FS% and EF%), followed by an increase in the left ventricular systolic dimension (Figures 2A–C). Systolic dysfunction was noted in two doxorubicin-treated monkeys as early as days 39–56 but was clearly evident in 3/12 monkeys by day 67. Between study days 67 and 127, all doxorubicin-treated monkeys exhibited systolic failure [reduced fractional shortening (Figure 2A), ejection fraction (Figure 2B) and increased left ventricular systolic diameter (Figure 2C)]. Over the last four assessment intervals (Study Days 91, 105, 116, and 127), mean fractional shortening progressively decreased by 26–54%, and mean ejection fraction progressively decreased by 18–39% in doxorubicin-treated monkeys compared with time-matched controls.

**Table 3:** Summary of echocardiographic and telemetry measurements following doxorubicin treatment in cynomolgus monkey.

		**Day of study**					
**Parameter**	**Treatment**	**0**	**15-18**	**36-39**	**56-63**	**91-92**	**Final**
Ejection fraction	Vehicle	0.82 ± 0.08	0.79 ± 0.04	0.82 ± 0.05	0.80 ± 0.06	0.82 ± 0.05	0.80 ± 0.06
	Doxorubicin	0.84 ± 0.07	0.80 ± 0.05	0.79 ± 0.10	0.76 ± 0.06	0.67 ± 0.16^*^	0.48 ± 0.12
Fractional shortening	Vehicle	0.42 ± 0.08	0.38 ± 0.04	0.41 ± 0.05	0.39 ± 0.06	0.40 ± 0.06	0.39 ± 0.06
	Doxorubicin	0.43 ± 0.07	0.39 ± 0.05	0.39 ± 0.09	0.35 ± 0.05^*^	0.30 ± 0.11^*^	0.18 ± 0.06
LVDs (cm)	Vehicle	1.04 ± 0.19	1.09 ± 0.14	1.08 ± 0.13	1.14 ± 0.17	1.11 ± 0.19	1.15 ± 0.18
	Doxorubicin	0.98 ± 0.20	1.10 ± 0.19	1.13 ± 0.21	1.20 ± 0.20	1.39 ± 0.37	1.64 ± 0.25
LV contractility (dP/dtmax)	Vehicle	2,309 ± 477	2,859 ± 872	2,815 ± 816	3,304 ± 1,019	2,866 ± 955	2,746 ± 693
	Doxorubicin	2,710 ± 894	2,368 ± 702	2,531 ± 745	2,706 ± 923	2,088 ± 417	1,812 ± 360
QTc (msec)	Vehicle	276 ± 27	277 ± 29	276 ± 25	269 ± 25	267 ± 28	271 ± 25
	Doxorubicin	299 ± 26	324 ± 25^*^	321 ± 26^*^	313 ± 27^*^	331 ± 32^*^	350 ± 49
Heart rate (bpm)	Vehicle	106 ± 11	120 ± 23	116 ± 19	114 ± 19	115 ± 21	111 ± 19
	Doxorubicin	112 ± 17	113 ± 17	121 ± 17	115 ± 20	133 ± 25	138 ± 28
Mean BP (mm Hg)	Vehicle	70 ± 12	76 ± 13	76 ± 14	76 ± 13	73 ± 13	75 ± 15
	Doxorubicin	76 ± 13	77 ± 15	77 ± 16	75 ± 17	69 ± 18^*^	66 ± 20

*All values are means ± SD (N = 12). Final = last available measurement prior to scheduled or unscheduled sacrifice*.

* *Statistically different from vehicle treated animals (p < 0.05). As Final was on different days for different animals, no statistical assessment is denoted*.

### Onset of Heart Failure: Telemetry Endpoints

**Figure 3:**
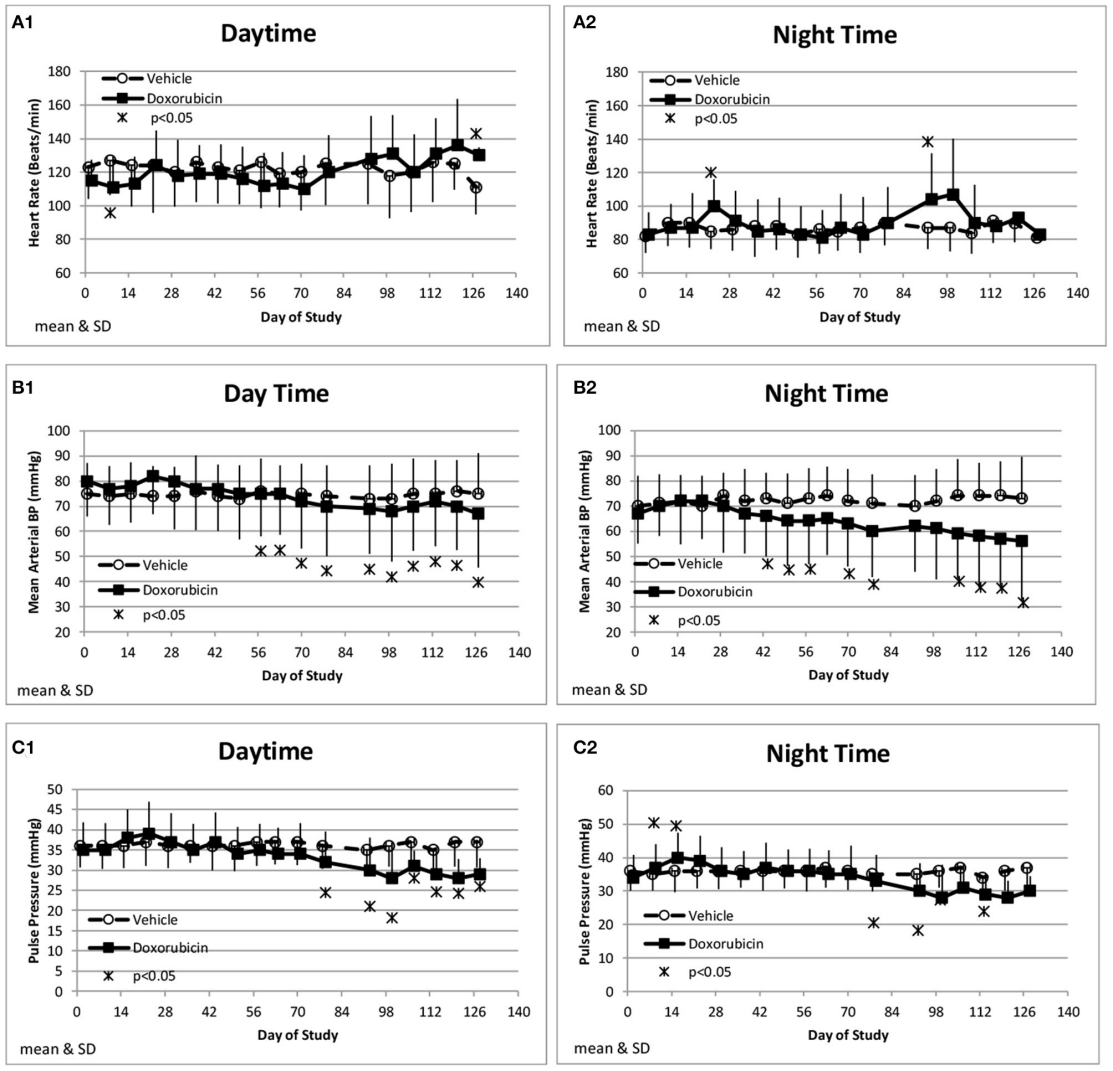
Hemodynamic changes. Changes in heart rate **(A)**, Mean Blood Pressure **(B)**, and Pulse pressure **(C)** averaged over the day (1) and night (2) time frames over the course of the study.

**Figure 4:**
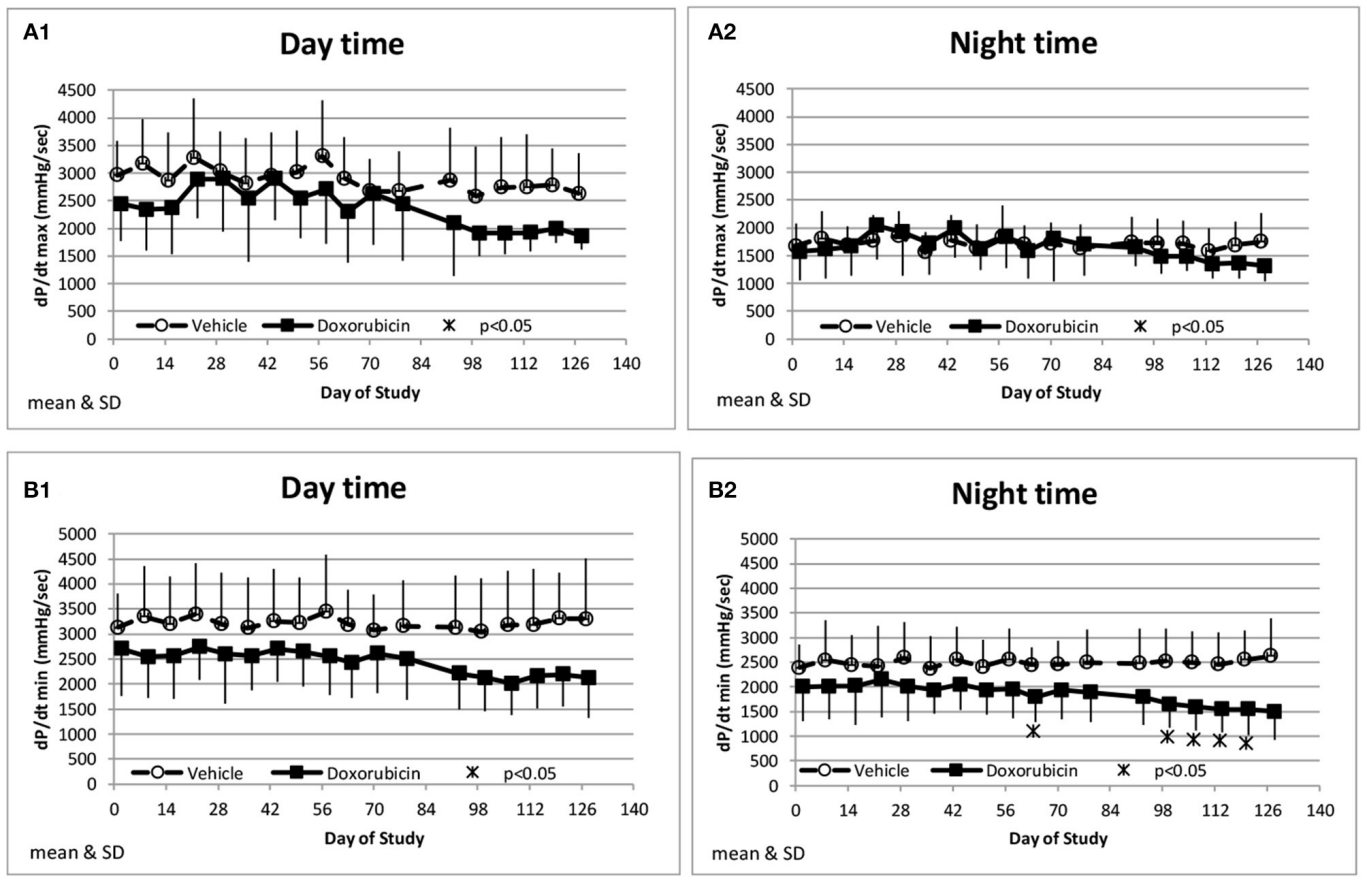
Measurements of indices of cardiac contractility. Changes in dP/dtmax during the day and night cycle times **(A1,A2)**, and dP/dtmin **(B1,B2)** averaged over the day and night time frames over the course of the study.

Along with the decrease in cardiac contractility, doxorubicin progressively lowered mean arterial pressure (Table 4). Mean arterial pressure was significantly lower in the light and dark cycles from Study Days 43 through 127 by 7–17 mmHg (−10 to −23%) (Figures 3B1,B2). Arterial pulse pressure (the difference between systolic and diastolic pressure) was significantly lower by 4–9 mmHg (−11 to −24%) in doxorubicin-treated monkeys beginning around Study Days 78–92 through the end of the dosing phase (Figures 3C1,C2), indicating a decrease in arterial stiffness. Doxorubicin had no effect on left ventricular end diastolic pressure (data not shown). Beginning on Study Day 92 through the end of the dosing phase, dP/dtmin values were lower in the dark cycle by 881–1,125 mmHg/s (−35 to −43%) in doxorubicin-treated animals compared with controls, with statistical significance noted at most time points; a similar pattern and magnitude was evident in the light cycle but did not reach statistical significance (Figure 4, Table 4). The dP/dtmax values trended lower in doxorubicin-treated monkeys by 665–824 mmHg/s (−26 to −30%) in the light cycle from Study Days 92 through 127; a similar pattern was evident in the dark cycle beginning on Study Day 99 but with a smaller magnitude.

**Table 4A:** Relationship between contractile function, cardiac biomarkers, QTc interval and myocyte injury in NHP treated chronically with doxorubicin.

**NHP#**	**Dox (mg/kg)**	**Telemetry unit type**	Functional effect or endpoint impacted^*^					
			**dP/dT Max**	**EF**	**QTc interval**	**cTnI**	**NT-pro-BNP**	**Cardiac injury**
104928	16	BP + LVP						Minimal (U)
104929	13	BP + LVP						(S)
104930	16	BP + LVP						Mild (U)
104931	16	BP + LVP						Minimal (S)
104932	11	BP + LVP						Mild (U)
104933	17	BP + LVP						(U)
104934	13	BP	ND					(U)
104935	17	BP	ND					(U)
104936	13	BP	ND					Mild (U)
104937	16	BP	ND		ND			Mild (U)
104938	13	BP	ND					(U)
104939	13	BP	ND					Minimal (U)

* *Cardiac contractility (dP/dtmax derived from LVP): >10% reduction = red; <10% = green. Ejection fraction (EF): >10% reduction = red; <10% = green. QTc interval: >10% prolongation = red; <10% prolongation = green. Troponin (cTnI): ≥2 samples with signal (>0.05 ng/ml) = red; 1/0 samples with signal = green. Natriuretic peptide (NT-pro-BNP): ≥2 samples with signal (>0.05 ng/ml) = red; no signal = green. Cardiac injury: presence of vacuolation = red; no vacuolation = green*.

*S, Scheduled sacrifice; U, Unscheduled sacrifice*.

*ND, not determined due to lack of LVP instrumentation or ECG failure*.

**Table 4B:** Relationship between contractile function, cardiac biomarkers, QTc interval and myocyte injury in NHP treated chronically with Vehicle.

**NHP#**	**Dose (mg/kg)**	**Telemetry unit type**	Functional effect or endpoint impacted^*^					
			**dP/dT Max**	**EF**	**QTc interval**	**cTnI**	**NT-pro-BNP**	**Cardiac injury**
104916	0	BP + LVP						(S)
104917	0	BP + LVP						(S)
104918	0	BP + LVP						(S)
104919	0	BP + LVP						(S)
104920	0	BP + LVP						(S)
104921	0	BP	ND					(S)
104922	0	BP	ND					(S)
104923	0	BP	ND		ND			(S)
104924	0	BP	ND					(S)
104925	0	BP	ND					(S)
104926	0	BP	ND					(S)
104927	0	BP	ND					(S)

* *Cardiac contractility (dP/dtmax derived from LVP): >10% reduction = red; no effect (<10%) = green. Ejection fraction (EF): >10% reduction = red; no effect (<10%) = green. QTc interval: >10% prolongation = red; no effect (<10% prolongation) = green. Troponin (cTnI): ≥2 samples with signal (>0.05 ng/ml) = red; 1/0 samples with signal = green. Natriuretic peptide (NT-pro-BNP): ≥2 samples with signal (>0.05 ng/ml) = red; no signal = green; excluded as signal was observed at baseline (pre-dose phase) = gray. Cardiac injury: presence of vacuolation = red; no vacuolation = green*.

*S, Scheduled sacrifice; U, Unscheduled sacrifice*.

*ND, not determined due to lack of LVP instrumentation or ECG failure*.

*No histopathological evidence of cardiac injury was seen in any of the vehicle treated animals and all were sacrificed as scheduled*.

**Figure 5:**
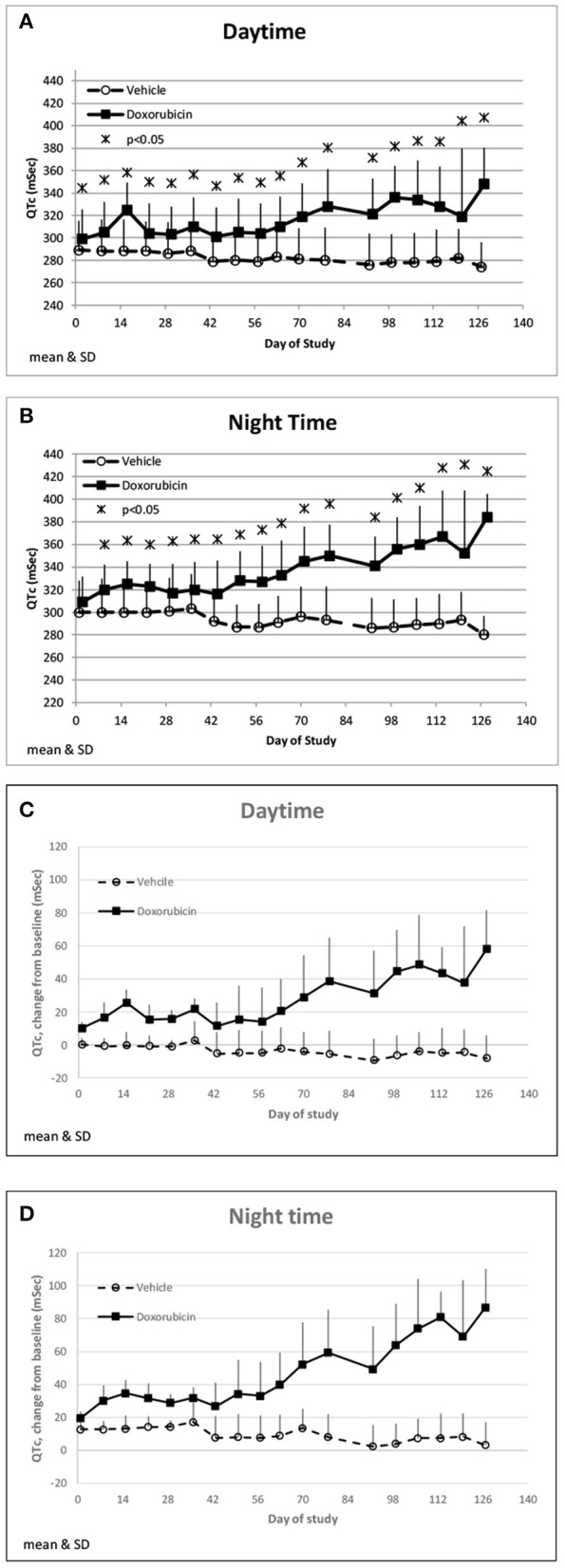
QTc changes. Changes in QTc averaged over the day **(A)** and night **(B)** time frames over the course of the study. Changes in QTc from baseline averaged over the day **(C)** and night **(D)** time frames over the course of the study.

Doxorubicin treatment had no effect on sinoatrial (PR interval) or ventricular (QRS interval) conduction times (data not shown), but significantly and progressively cause delayed ventricular repolarization (QTc intervals) that was observed during the day and night data collection time frames (Figures 5A,B). The QTc interval was significantly (>10 ms) prolonged from baseline in doxorubicin-treated monkeys after study day 50. During the dark cycle, the QTc interval was prolonged by 41–104 ms (14–37%; days 50–127). During the light cycle, the QTc interval was prolonged by 38–74 ms (14–27%; days 70–127). Heart rate was mildly higher after day 90 by 11–19 bpm (9–17%) (Table 4, Figures 3A1,A2, 5A–D).

### Cardiac Biomarkers

**Figure 6:**
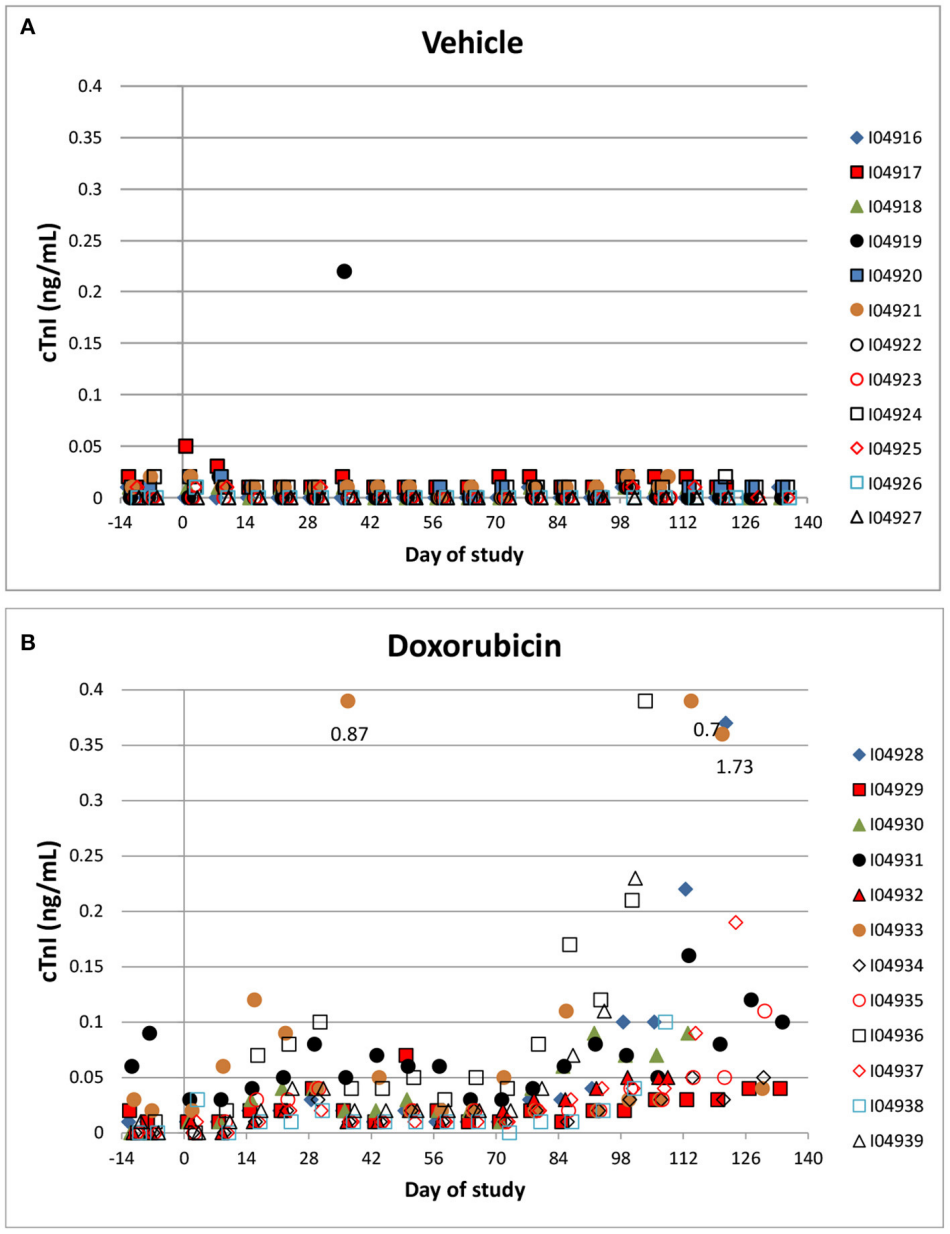
Cardiac Troponin. Changes in individual animal cTnI in control **(A)** a treated **(B)** animals over the course of the study. Filled markers denote animals equipped with LVP catheters.

Troponin (cTnI) levels were increased slightly above baseline values in 8/12 animals administered doxorubicin at one or more intervals. Most of the increases occurred after day 60 of the dosing phase (Figures 6A,B). In the control group, cTnI levels were consistently low (<0.05 ng/mL) with only a single excursion above 0.2 ng/mL (day 35). Based on qualitative assessment, the surgical insertion and sustained presence of a transmural left ventricular pressure catheter had no impact on cTnI levels.

**Figure 7:**
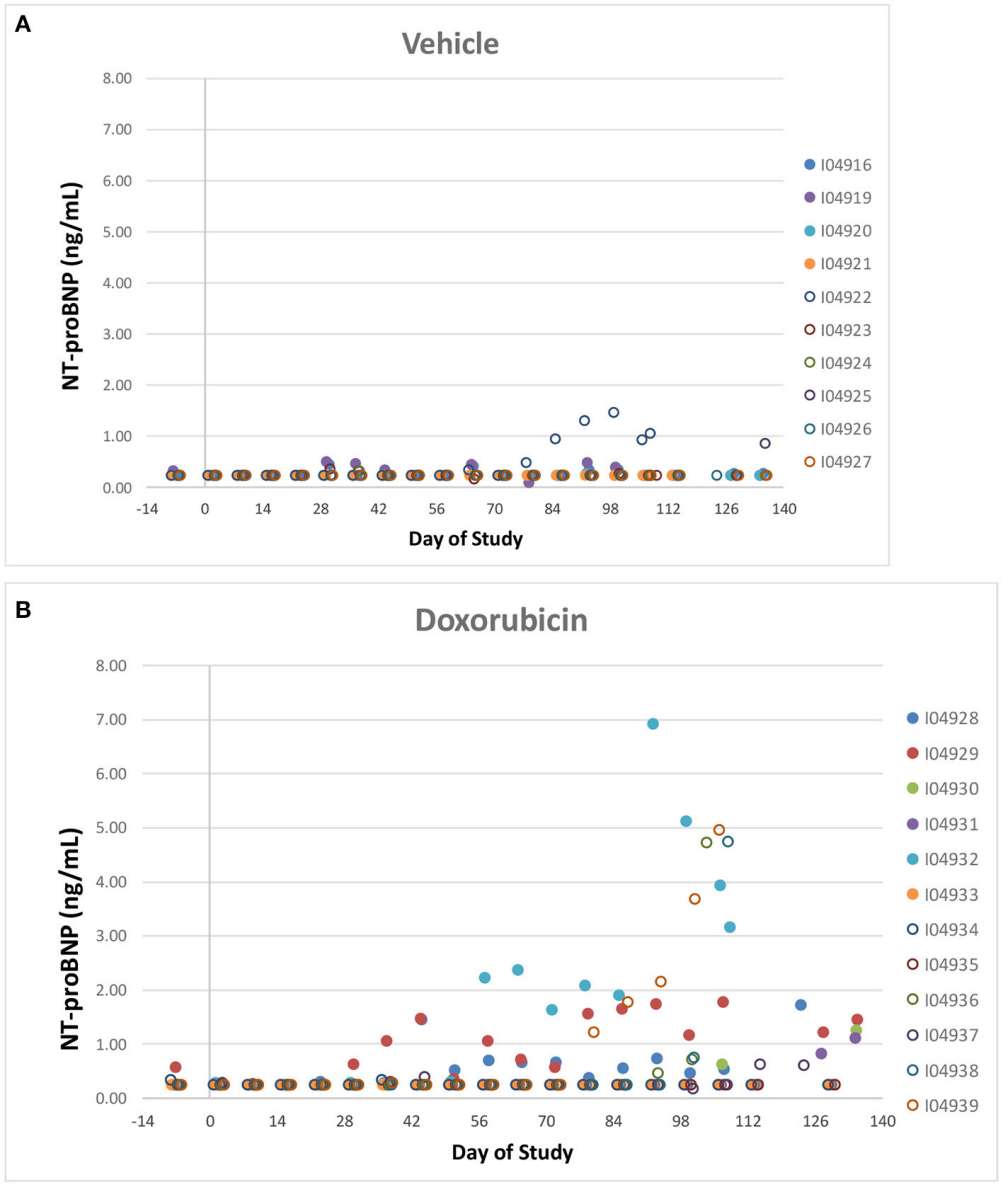
NT-proBNP. Individual animal NT-proBNP measurements in control **(A)** a treated **(B)** animals over the course of the study. Filled markers denote animals equipped with LVP catheters.

Two vehicle treated animals had elevated NT-proBNP over the course of the study (including pre-dose) with no other indication of heart failure (e.g., normal EF%) and that data was therefore excluded. NT-proBNP was increased (>1 ng/mL) in several treated animals (8/12) over the course of the study while only one of the remaining ten vehicle treated animal exceeded 1 ng/mL (Figures 7A,B). There was no evidence of any impact of the instrumentation on the NT-proBNP levels.

**Figure 8:**
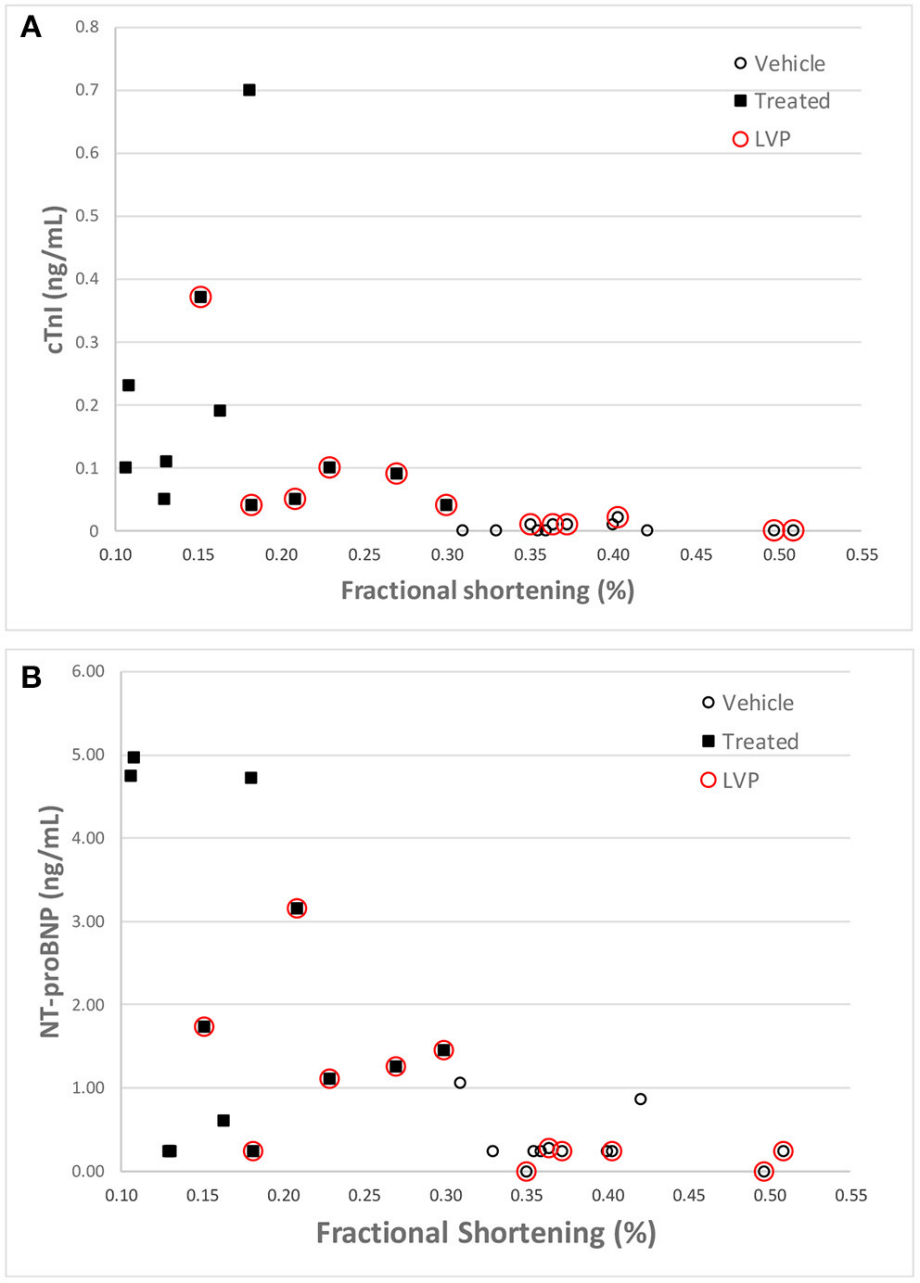
Correlation of cTnI **(A)** and NT-proBNP **(B)** levels with Fractional Shortening measurements at the final echocardiography measurement for each animal. Red circles denote animals equipped with LVP catheters.

Like cTnI, NT-Pro-BNP levels where elevated in some doxorubicin-treated animals after day 60, a time frame that coincided with onset of heart failure. Plots of final biomarker levels vs. EF% and FS% was created to examine the relationship of cardiac biomarkers level with the degree of heart failure, but no clear correlation was observed (Figures 8A,B).

### Myocardial Histopathology

**Figure 9:**
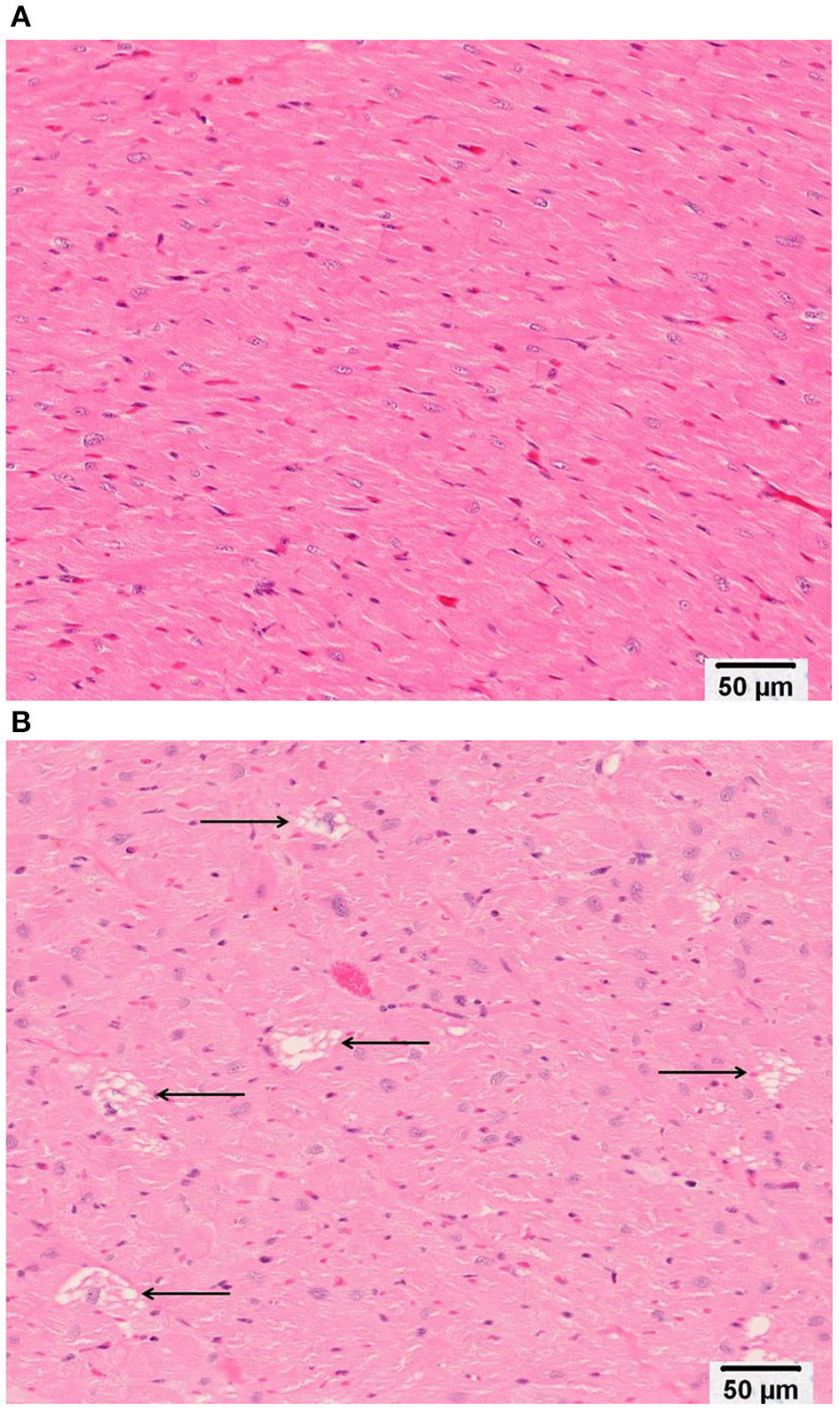
Representative photomicrographs of heart tissue from **(A)** a Vehicle treated animal with normal myocardium; and **(B)** Doxorubicin treated animals with multifocal, cytoplasmic vacuolar degeneration of cardiomyocytes (arrows). Bar equals 50 microns.

Minimal (three of 12 animals) to mild (four of 12 animals), multifocal, vacuolar degeneration of cardiomyocytes was observed in seven of 12 animals receiving doxorubicin and zero of 12 animals receiving vehicle (Tables 4A, 4B, Figure 9). Cytoplasmic vacuolation of cardiomyocytes was observed more often in animals that were euthanized in a moribund condition compared with those that survived to scheduled termination. Light microscopic evidence of cytoplasmic vacuolation of cardiomyocytes was consistently associated with elevation of cTnI (six of seven NHP with cardiac injury), however, elevation of cTnI was not uniformly associated with cardiac injury (Table 4B: see NHP #104933 and 104935).

## Discussion and Conclusions

This study successfully documented the time course of LVD following chronic treatment with doxorubicin, which clearly indicates that heart failure can be recapitulated in the cynomolgus monkey. Left ventricular contractile function was assessed with both direct measurement of left ventricular pressure and echocardiography, and telemetry measurements were used to monitor hemodynamics and cardiac conduction and repolarization over the course of the study. Cardiac biomarkers were also tracked to monitor cardiac damage (cTnI) and ventricular wall stress (NT-proBNP). Finally, cardiac tissues were examined to determine the incidence of histopathological changes. The integrated use of these cardiovascular endpoints highlight their utility and value in detecting drug-induced LVD and heart failure in this species and validates their use for cardiovascular safety assessment.

Echocardiography is a common clinical method used to measure doxorubicin-related cardiotoxicity, which has a slow onset time in human (25–27). the current study confirms that long-term treatment with doxorubicin caused cardiac dysfunction in all treated animals, and myocyte degeneration (confirmed by histopathology) was observed in a portion of cynomolgus monkeys, as in humans (28). The progressive nature of doxorubicin-induced heart failure with reduced ejection fraction (and fractional shortening) in monkeys (EF: 20–25%) is consistent with clinical phenotype and delayed onset observed in patients (25–27, 29).

In our study, reductions in ejection fraction and fractional shortening were also reflected by trends toward decreases in left ventricular dP/dt_max_ (decreased inotropy) and dP/dt_min_ (decreased lusitropy) (Figure 4). LVP telemetry is an invasive method but it is a sensitive technique for the continual measurement of cardiac contractility in free-moving unanesthetized animals (12). Thus, it avoids the confounding influence of anesthetic sedation and/or restraint that is required to perform echocardiography in monkeys. The lack of functional or biomarker differences between the animals instrumented for blood pressure only vs. blood and ventricular pressure instrumentation suggests that a transmural ventricular catheter did not impact the negative inotropic response to doxorubicin. Doxorubicin has been shown to decrease left ventricular contractility in other species (dogs) both acutely (17) and after chronic treatment (30). The changes in cardiac function were accompanied by enlargement of left ventricular systolic diameter. The increase in left ventricular diameter during systole in this study is consistent with the observed decrease in ejection fraction and fractional shortening. Interestingly, the left ventricular diastolic diameter did not markedly increase in the same time frame, and therefore the decrease in function was not accompanied by signs of cardiac hypertrophy as has been reported with other models (7, 31).

Troponin release (cTnI) is a marker of cardiac myocyte damage and is useful clinically for diagnosing cardiac damage resulting from myocardial infarction. Previous studies in rats (14, 32) and dogs (33) have demonstrated doxorubicin-related cTnI increases. However, in this study, while individual animals had sporadic increases in cTnI, there were no consistent changes. The incidence and magnitude of increases in cTnI was greater toward the end of the study, concurrent with changes indicating decreased cardiac function. It is possible that the gradual onset of the compromise in cardiac function did not cause sufficient ongoing myocyte damage to result in sustained increases in cTnI. The limits of detection for the assay used in this study have been superseded by newer high- and ultra-high sensitivity assays and it is possible that more consistent treatment-related small changes in cTnI could be detected by those assays (32, 34).

Naturetic peptides (BNP and NT-proBNP) are considered to be the “gold standard” for diagnosis of heart failure in the clinic (35, 36) with NT-proBNP preferred by some due to a longer persistence in the blood. NT-proBNP has been used clinically to predict cardiac damage associated with anthracycline treatment (37). In this study, two control animals had consistently elevated NT-proBNP levels for much of the study (however levels were reduced at the end of the study). As these animals had normal left ventricular function over this time frame, the underlying reason for this elevation is unknown and has been excluded. Both cTnI and NT-proBNP were occasionally but not consistently higher at the end of the study in animals with LVD. The reason for the lack of good correlation is not known. NT-proBNP has rarely been measured in the NHP though increases have been reported in animals treated with an immune checkpoint inhibitor (38). The observed progressive increase in QTc in the treated animals is likely due to structural changes in the myocardium (minimal to mild degeneration of cardiomyocytes) of affected animals. Similar changes have been observed in human clinical studies with doxorubicin and other cancer therapeutics. However, treatment-related QTc increases have been observed with no concomitant changes in ejection fraction (10, 39). The underlying mechanisms for the QTc change, which are specifically not related to direct or indirect hERG channel blockade (40) are unclear, but likely reflect electric remodeling and loss of repolarization reserve secondary to doxorubicin-induced heart failure.

Blood pressure decreased slightly, and heart rates tended to increase in treated animals after week 43. Although the mechanism of these changes is not clear, they may be related to the changes in sympathetic tone concurrent with the decline in cardiac function.

Other studies have demonstrated that doxorubicin can cause detrimental effects to mitochondria and the sarcoplasmic reticulum (41). This histopathological analysis in this study was limited to light microscopy, where there was only minimal to mild vacuolation of cardiomyocytes (seven of 12 treated animals). Overall, based on the study results, dramatic cardiovascular functional changes seen in the doxorubicin-treated animals were not accompanied by severe histopathological changes.

The original dose selected, 2 mg/kg/week, caused bone marrow toxicity that resulted in progressively decreased neutrophil counts. A dosing holiday for all the animals allowed the neutrophil counts to recover. The subsequent 1 mg/kg every other week dosing regimen spared neutrophil counts but was insufficient to degrade cardiac function. An increase in dose to 1 mg/kg/week resulted in generally progressive decreases in ejection fraction and fractional shortening. There was also evidence of variable response to treatment in that some animals required additional dosing holidays (due to decreased neutrophil counts) while others tolerated the weekly dosing schedule until study termination.

This study demonstrated the ability to detect progressive changes in cardiac function related to heart failure using telemetry and echocardiography in the cynomolgus monkey administered clinically relevant levels of the anthracycline oncology agent doxorubicin. This study demonstrated the utility and sensitivity of serial echocardiography to detect decreases in cardiac function. These echocardiographic changes were generally accompanied in more than half of the doxorubicin treated animals by changes in clinical biomarkers such as cTnI and NT-proBNP concentrations and occasional histopathological lesions. This type of adaptive study design, where doses were modified based on monitored clinical pathology endpoints is potentially more relevant for study of the impact of interventions designed to ameliorate toxicity related to anthracycline treatment which has been advocated by others (42). This required flexibility illustrates the difficulty of modeling the clinical liability of late or delayed decreases in cardiac function with a standard preclinical study design. The cardiac liability is only replicated with a dosing paradigm that avoids overt toxicity while maintaining a high enough dose to produce cardiac toxicity. The serial measurements of cardiac function presented here provided a unique documentation of the progressive decreases in cardiac function during heart failure in the NHP after repeated administration of doxorubicin.

## Data Availability Statement

The raw data supporting the conclusions of this article will be made available by the authors, without undue reservation.

## Ethics Statement

All research protocols were approved by the Testing Facility Institutional Animal Care and Use Committees (IACUC) prior to dose administration.

## Author Contributions

All authors listed have made a substantial, direct and intellectual contribution to the work, and approved it for publication.

## Conflict of Interest

All authors were employed by Amgen, Inc. NE is employed by Seattle Genetics. The authors declare that the research was conducted in the absence of any commercial or financial relationships that could be construed as a potential conflict of interest.

## References

[1] Bristow MR Billingham ME Mason JW Daniels JR. Clinical spectrum of anthracycline antibiotic cardiotoxicity. Cancer Treat Rep. (1978) 62:873–9. 667861

[2] Shan K Lincoff AM Young JB. Anthracyline-Induced Cardiotoxicity. Ann Intern Med. (1996) 125:47–58. 10.7326/0003-4819-125-1-199607010-00008 8644988

[3] Zang S Liu X Bawa-Khalfe T Lu L Yi LL Liu LF. Identification of the molecular basis of doxorubicin-induced cardiotoxicity. Nat Med. (2012) 18:1639–45. 10.1038/nm.2919 23104132

[4] Montaigne D Hurt C Neviere R. Mitochondria death/survival signaling pathways in cardiotoxicity induced by anthracyclines and anticancer-targeted therapies. Biochem Res Int. (2012) 2012:951539. 10.1155/2012/951539 22482055 PMC3318211

[5] Takahashi SS Denvir MA Harder L Miller DJ Cobbe SM Kawakami M. Effects of in vitro and in vivo exposure to doxorubicin (adriamycin) on caffeine-induced Ca2+ release from sarcoplasmic reticulum and contractile protein function in ‘chemically-skinned'rabbit ventricular trabeculae. Jap J Pharm. (1998) 76:405–13. 10.1254/jjp.76.405 9623719

[6] Minotti G Menna P Salvatorelli E Cairo G Gianni L. Anthracyclines: molecular advances and pharmacologic developments in antitumor activity and cardiotoxicity. Pharmacol Rev. (2004) 56:185–229. 10.1124/pr.56.2.6 15169927

[7] Ewer MS Lippman SM. Type II chemotherapy-related cardiac dysfunction: time to recognize a new entity. J Clin Oncol. (2005) 23:2900–2. 10.1200/JCO.2005.05.827 15860848

[8] Eschenhagen T Force T Ewer MS De Keulenaer GW Suter TM Anker SD. Cardiovascular side effects of cancer therapies: a position statement from the heart failure association of the european society of cardiology. Eur J Heart Fail. (2011) 13:1–10. 10.1093/eurjhf/hfq213 21169385

[9] Sparano JA. Doxorubicin/Taxane combinations: cardiac toxicity and pharmacokinetics. Semin Oncol. (1999) 26:14–9. 10426454

[10] Nousiainen T Vanninen E Rantala A Jantunen E Hartikainen J. QT dispersion and late potentials during doxorubicin therapy for non-Hodgkin's lymphoma. J Intern Med. (1999) 245:359–64. 10.1046/j.1365-2796.1999.00480.x 10356598

[11] Lee BH Goodenday LS Muswick GJ Yasnoff WA Leighton RF Skell RT. Alterations in left ventricular diastolic function with doxorubicin therapy. J Am Coll Cardiol. (1987) 9:184–8. 10.1016/S0735-1097(87)80099-2 3794095

[12] Guth BD Chiang AY Doyle J Engwall MJ Guillon JM Hoffmann P. The evaluation of drug-induced changes in cardiac inotropy in dogs: Results from a HESI-sponsored consortium. J Pharmacol Toxicol Methods. (2015) 75:70–90. 10.1016/j.vascn.2015.02.002 25843226

[13] Pugsley MK Guth B Chiang A Doyle J Engwall M Guillon JM. A HESI consortium update on cardiac contractility endpoints. J Pharmacol Toxicol Methods. (2016) 100:390–1. 10.1016/j.vascn.2016.02.180

[14] Cove-Smith L Woodhouse N Hargreaves A Kirk J Smith S Price SA. An integrated characterization of serological, pathological, and functional events in doxorubicin-induced cardiotoxicity. Toxicol Sci. (2014) 140:3–15. 10.1093/toxsci/kfu057 24675088

[15] Migrino RQ Aggarwal D Konorev E Brahmbhatt T Bright M Kalyanaraman B. Early detection of doxorubicin cardiomyopathy using two-dimensional strain echocardiography. Ultrasound Med Biol. (2008) 34:208–14. 10.1016/j.ultrasmedbio.2007.07.018 17935867 PMC2582214

[16] Hanai K Takabe K Manabe S Nakano M Kohda A Matsuo M. Evaluation of cardiac function by echocardiography in dogs treated with doxorubicin. J Toxicol Sci. (1996) 21:1–10. 10.2131/jts.21.1 8852283

[17] Ditchey RV LeWinter MM Higgins CB. Acute effects of doxorubicin (adriamycin) on left ventricular function in dogs. Int J Cardiol. (1984) 6:341–50. 10.1016/0167-5273(84)90194-3 6480163

[18] Manno RA Grassetti A Oberto G Nyska A Ramot Y. The minipig as a new model for the evaluation of doxorubicin-induced chronic toxicity. J Appl Toxicol. (2016) 36:1060–72. 10.1002/jat.3266 26614124

[19] Sieber SM Correa P Young DM Dalgard DW Adamson RH. Cardiotoxic and possible leukemogenic effects of adriamycin in nonhuman primates. Pharmacology. (1980) 20:9–14. 10.1159/000137337 6769133

[20] Malik M Allison DE Dybdal N Lum BL. Trastuzumab (recombinant humanized monoclonal anti-HER2) clearance is not altered in a non-clinical model of doxorubicin-induced cardiotoxicity. Proc Am Assoc Cancer Res. (2004) 45 (Suppl):1245.

[21] Takayama S Thorgeirsson UP Adamson RH. Chemical carcinogenesis studies in nonhuman primates. Proc Japan Acad Series B. (2008) 84:176–88. 10.2183/pjab.84.176 PMC3665368 18941297

[22] Heyen J Vargas H. The use of nonhuman primates in cardiovascular safety assessment. In: Bluemel J. Korte S Schenck E Weinbauer G, editors. The Nonhuman Primate in Nonclinical Drug Development and Safety Assessment. San Francisco, CA: Academic Press (2015) 551–78. 10.1016/B978-0-12-417144-2.00029-9

[23] Clark JD Gebhart GF Janet CG Keeling ME Kohn DF. The 1996 guide for the care and use of laboratory animals. ILAR J. (1997) 38:41–48. 11528046 10.1093/ilar.38.1.41

[24] Food and Drug Administration. Guidance for Industry: Estimating the Maximum Safe Starting Dose in Initial Clinical Trials for Therapeutics in Adult Healthy Volunteers. Beltsville, MD: Center for Drug Evaluation and Research (CDER) (2005) 1245.

[25] Parisi AF Moynihan PF Folland ED. Echocardiographic evaluation of left ventricular function. Med Clin North Am. (1980) 64:61–81. 10.1016/S0025-7125(16)31625-X 6987470

[26] Steinherz LJ Steinherz PG Tan CT Heller G Murphy ML. Cardiac toxicity 4 to 20 years after completing anthracycline therapy. JAMA. (1991) 266:1672–7. 10.1001/jama.1991.03470120074036 1886191

[27] Lipshultz SE Lipsitz SR Sallan SE Dalton VM Mone SM Gelber RD. Chronic progressive cardiac dysfunction years after doxorubicin therapy for childhood acute lymphoblastic leukemia. J Clin Oncol. (2005) 23:2629–36. 10.1200/JCO.2005.12.121 15837978

[28] Isner JM Ferrans VJ Cohen SR Witkind BG Virmani R Gottdiener JS. Clinical and morphologic cardiac findings after anthracycline chemotherapy: analysis of 64 patients studied at necropsy. Am. J. Card. (1983) 51:1167–1174. 6573121 10.1016/0002-9149(83)90364-8

[29] Geisberg CA Sawyer DB. Mechanisms of anthracycline cardiotoxicity and strategies to decrease cardiac damage. Curr Hypertens Rep. (2010) 12:404–10. 10.1007/s11906-010-0146-y 20842465 PMC3999517

[30] Astra LI Hammond R Tarakji K Stephenson LW. Doxorubicin-Induced Canine CHF. J Card Surg. (2003) 18:301–6. 10.1046/j.1540-8191.2003.02032.x 12869174

[31] Toyoda Y Okada M Kashem MA. A canine model of dilated cardiomyopathy induced by repetitive intracoronary doxorubicin administration. J Thorac Cardiovasc Surg. (1998) 115:1367–73. 10.1016/S0022-5223(98)70221-1 9628680

[32] Reagan WJ York M Berridge B Schultze E Walker D Pettit S. Comparison of cardiac troponin I and T, including the evaluation of an ultrasensitive assay, as indicators of doxorubicin-induced cardiotoxicity. Toxicol Pathol. (2013) 41:1146–58. 10.1177/0192623313482056 23531791

[33] Burgener IA Kovacevic A Mauldin GN Lombard CW. Cardiac troponins as indicators of acute myocardial damage in dogs. J Vet Internal Med. (2006) 20:277–83. 10.1111/j.1939-1676.2006.tb02857.x 16594583

[34] Herman EH Knapton A Rosen E Thompson K Rosenzweig B Estis J. A multifaceted evaluation of imatinib-induced cardiotoxicity in the rat. Toxicolic Pathol.(2011) 39:1091–106 10.1177/0192623311419524 21937741

[35] Maisel AS Choudhary R. Biomarkers in acute heart failure—state of the art. Nat Rev Cardiol. (2012) 9:478–90. 10.1038/nrcardio.2012.60 22547172

[36] Latini R Masson S. NT-PROBNP: a guide to improve the management of patients with heart failure. EJIFCC. (2013) 24:78–84. 27683441 PMC4975180

[37] Romano S Fratini S Ricevuto E Procaccini V Stifano G. Serial measurements of NT-proBNP are predictive of not-high-dose anthracycline cardiotoxicity in breast cancer patients. Br J Cancer. (2011) 105:1663. 10.1038/bjc.2011.439 22068815 PMC3242597

[38] Ji C Roy MD Golas J Vitsky A Ram S Kumpf SW. Myocarditis in cynomolgus monkeys following treatment with immune checkpoint inhibitors. Clin Cancer Res. (2019) 25:4735–48. 10.1158/1078-0432.CCR-18-4083 31085720

[39] Curigliano G Mayer EL Burstein HJ Winer EP Goldhirsch A. Cardiac toxicity from systemic cancer therapy: a comprehensive review. Prog Cardiovasc Dis. (2010) 53:94–104. 10.1016/j.pcad.2010.05.006 20728696

[40] Vargas HM Bass AS Koerner J Matis-Mitchell S Pugsley MK Skinner M. Evaluation of drug-induced QT interval prolongation in animal human studies: a literature review of concordance. Br J Pharmacol. (2015) 172:4002–11. 10.1111/bph.13207 26031452 PMC4543608

[41] Zhou S Starkov A Froberg MK Leino RL Wallace KB. Cumulative and irreversible cardiac mitochondrial dysfunction induced by doxorubicin. Cancer Res. (2001) 61:771–7. 11212281

[42] Gianni L Herman EH Lipshultz SE Minotti G Sarvazyan N Sawyer DB. Anthracycline cardiotoxicity: from bench to bedside. J Clin Oncol. (2008) 26:3777–84. 10.1200/JCO.2007.14.9401 18669466 PMC3018290

